# Whole-body hypothermia in mild neonatal encephalopathy: protocol for a multicentre phase III randomised controlled trial

**DOI:** 10.1186/s12887-024-04935-4

**Published:** 2024-07-18

**Authors:** Reema Garegrat, Paolo Montaldo, Constance Burgod, Stuti Pant, Munirah Mazlan, Balamurugan Palanisami, Ela Chakkarapani, Kerry Woolfall, Samantha Johnson, Patricia Ellen Grant, Sarah Land, Mariam Mahmoud, Tony Brady, Victoria Cornelius, Eleri Adams, Jon Dorling, Narendra Aladangadi, Paul Fleming, Ronit Pressler, Andrew Shennan, Stavros Petrou, Aung Soe, Paul Basset, Seetha Shankaran, Sudhin Thayyil

**Affiliations:** 1 Centre for Perinatal Neuroscience, Department of Brain Sciences, Imperial College London, Du Cane Road, London, W12 0HS England; 2 Department of Woman, Child, and General and Specialized Surgery, University of Campania “Luigi Vanvitelli”, Naples, Italy; 3 Neonatal Medicine, Liverpool Women’s NHS Foundation Trust, Liverpool, UK; 4 University of Bristol and St Michaels Hospital NHS Trust, Bristol, UK; 5 Public Health, Policy and Systems, University of Liverpool, Liverpool, UK; 6 Department of Population Health Sciences, University of Leicester, Leicester, UK; 7 Departments of Radiology and Pediatrics, Boston Children,s Hospital, Harvard Medical School, Boston, USA; 8 PEEPS HIE Charity, London, UK; 9 Parent representative, London, UK; 10 Stats Consultancy, London, UK; 11 Sealed Envelope, London, USA; 12 Imperial Clinical Trials Unit, Imperial College London, London, UK; 13 Neonatal Medicine, John Radcliffe Hospital NHS Trust, London, UK; 14 Neonatal Medicine, University Hospital Southampton NHS Trust, London, UK; 15 Neonatal Medicine, Homerton Healthcare NHS Foundation Trust, London, UK; 16 Department of Neurophysiology, Great Ormond Street Hospital, London, UK; 17 Department of Obstetrics, Kings College London, London, UK; 18 Professor of Health Economics, University of Oxford, Oxford, UK; 19 Oliver Fisher Neonatal Intensive Care Unit, Medway Maritime Hospital, Medway NHS Foundation Trust, Kent, UK; 20 Department of Neonatal-Perinatal Medicine, Wayne State University, Detroit, MI USA; 21 University of Texas at Austin, Dell Children’s Hospital, Austin, USA

**Keywords:** Mild encephalopathy, Hypothermia, Magnetic resonance spectroscopy

## Abstract

**Background:**

Mild hypoxic ischemic encephalopathy is associated with sub optimal cognition and learning difficulties at school age. Although whole-body hypothermia reduces death and disability after moderate or severe encephalopathy in high-income countries, the safety and efficacy of hypothermia in mild encephalopathy is not known. The cooling in mild encephalopathy (COMET) trial will examine if whole-body hypothermia improves cognitive development of neonates with mild encephalopathy.

**Methods:**

The COMET trial is a phase III multicentre open label two-arm randomised controlled trial with masked outcome assessments. A total of 426 neonates with mild encephalopathy will be recruited from 50 to 60 NHS hospitals over 2 ½ years following parental consent. The neonates will be randomised to 72 h of whole-body hypothermia (33.5 *±* 0.5 C) or normothermia (37.0 *±* 0.5 C) within six hours or age. Prior to the recruitment front line clinical staff will be trained and certified on expanded modified Sarnat staging for encephalopathy. The neurological assessment of all screened and recruited cases will be video recorded and centrally assessed for quality assurance. If recruitment occurs at a non-cooling centre, neonates in both arms will be transferred to a cooling centre for continued care, after randomisation. All neonates will have continuous amplitude integrated electroencephalography (aEEG) at least for the first 48 h to monitor for seizures. Predefined safety outcomes will be documented, and data collected to assess resource utilization of health care. A central team masked to trial group allocation will assess neurodevelopmental outcomes at 2 years of age. The primary outcome is mean difference in composite cognitive scores on Bayley scales of Infant and Toddler development 4th Edition.

**Discussion:**

The COMET trial will establish the safety and efficacy of whole-body hypothermia for mild hypoxic ischaemic encephalopathy and inform national and international guidelines in high income countries. It will also provide an economic assessment of whole-body hypothermia therapy for mild encephalopathy in the NHS on cost-effectiveness grounds.

**Trial registration number:**

NCT05889507 June 5, 2023.

## Introduction

Mild hypoxic ischemic encephalopathy (HIE) occurs in around 0.8 to 1 per 1000 livebirths [1]. In the UK approximately 1400 babies are admitted to neonatal units with HIE every year; of these around 600 have moderate or severe HIE and 800 have mild HIE [1].

Although in high income countries, neonates with mild HIE are unlikely to die or develop major neurodisability [2], careful long term follow-up studies have reported cognitive deficits that becomes apparent by 2 years and possibly increase with age. Finder et al. reported that the mean (SD) Bayley-III Cognitive Scale score of 55 un-cooled babies with mild HIE was 6 points lower than 152 healthy peers (98 [12] versus 104 [15]) [2] when assessed between 18 and 42 months of age, although only 68% could be assessed. Murray et al. reported mean IQ of 22 babies with mild HIE was 18 points lower than 30 healthy peers (99 versus 117; *p* < 0.001) at 5 years of age [3]. Moreover, 38% of children with mild HIE had special educational needs compared with 18% of their siblings and 0% of healthy peers [4]. The PRIME (Prospective Research in Mild Encephalopathy) study recruited 63 un-cooled babies with mild HIE from Canada, US, UK, and Thailand, of which 43 (68%) were assessed at 2 years; Seven (16%) had a Bayley Cognitive Scale Composite score of less than 85 points [5], indicating at least mild cognitive impairment. Given the higher occurrence of mild HIE, health and economic burden at a population level is likely to be substantial [6].

Whole-body hypothermia, an evidence-based therapy for babies with moderate or severe encephalopathy in high income countries [7], is increasingly used for babies with mild HIE without an adequate evaluation of safety and efficacy [8, 9]. In the UK, around 30% of the 3511 babies with mild hypoxic ischaemic encephalopathy admitted to neonatal units between 2011 and 2016 received whole-body hypothermia. During the same period, 830 neonates without encephalopathy were also cooled. Furthermore, the number of babies with moderate encephalopathy doubled from 141 to 293 indicating many neonates with mild HIE might have been misclassified as moderate HIE [10].

Separately, a London neonatal transport audit reported that of the 170 babies transported for whole-body hypothermia between 2017 and 2019, 45% had mild HIE or birth acidosis without encephalopathy and did not meet the current criteria for whole-body hypothermia [11].A structured neurological examination to determine severity of HIE prior to initiation of whole-body hypothermia was either not performed or not documented in most babies [11].

Short term outcomes on 7181 babies with mild HIE are available from the Canadian (*n* = 1089; cooled 36%) [12], Californian (*n* = 1364; cooled 71%) [13], Children’s Hospital Neonatal Consortium (*n* = 272; cooled 95%) [14]; US Children’s Hospitals National Database (*n* = 945; cooled 13%) [15] and the UK (*n* = 3511; cooled 30%) [16] registries. These data show that whole-body hypothermia significantly increased duration of ventilatory support (2 days versus 1 day), intensive care stay (9 days versus 6 days), need for invasive ventilation (60% versus 45%), use of opioid infusion (67% versus 12%), disseminated intravascular coagulation (8% versus 2%), hepatic dysfunction (23% versus 11%), cardiac dysfunction (8% versus 2%), discharge home on oxygen (26% vs. 15%) and tube feeding at hospital discharge (22% versus 13%) compared to usual care. Other adverse short-term outcomes noted only in babies with mild HIE who underwent whole-body hypothermia include hypotension (16%), thrombocytopenia (10%), coagulopathy (17%), persistent metabolic acidosis (8%), and subcutaneous fat necrosis (1%). No neurodevelopmental outcome data are available from any of these registries, so the long-term impact is unknown.

A well-designed observational study (COOL Prime Study: Comparative Effectiveness for Cooling Prospectively Infants with Mild Encephalopathy) evaluating the neurological outcomes of 430 neonates with mild HIE from 15 hospitals is currently ongoing in the USA (NCT04621279). However, in the absence of a randomised controlled arm, no conclusions about safety and efficacy of whole-body hypothermia can be made from observational data [17].

Lack of hypothermic neuroprotection and potential harms reported in two recent major hypothermia trials [18, 19] have highlighted the hazards of therapeutic drift, and the critical importance of conducting clinical trials before extending its use to untested populations. The first of these trials involved 408 neonates with moderate or severe HIE from low and middle-income countries (HELIX; Hypothermia for Encephalopathy in low and middle-income countries) [19], which reported a significant increase in mortality with whole-body hypothermia. The second trial (Preemie Hypothermia trial) included 151 premature neonates with moderate or severe HIE born between 33 + 0 to 35 + 5 weeks and reported that whole-body hypothermia increased the probably of death by 77% [18]. Unlike the original hypothermia trials where hyperthermia occurred in 14–29% of the control arms [20–22], both HELIX trial [19] and PREMIE hypothermia trials had much lower occurrence of hyperthermia in the control arm.

The British Association of Perinatal Medicine (BAPM) [23] and the American Academy of Pediatrics [16, 24, 25] have called for an urgent clinical trial to assess safety and efficacy of hypothermia for mild HIE.

### Aims

- To examine if whole-body hypothermia to 33.5 *±* 0.5 °C, initiated within six hours of birth and continued for 72 h, improves cognitive development at two years of age after mild neonatal encephalopathy when compared with normothermia at 37.0 *±* 0.5^0^C.
- To examine if a prospective trial-based economic evaluation supports the provision of whole-body hypothermia therapy for mild HIE in the NHS on cost-effectiveness grounds.

## Study design

### Setting

Cooling in mild encephalopathy (COMET) is a phase III multi-centre open label two-arm randomised controlled trial (RCT) with internal pilot and masked outcome assessments. Administration of cooling therapy cannot be masked. The trial will recruit from five neonatal operational delivery networks (ODN) involving around 50 to 60 NHS hospitals. The recruitment will occur at both tertiary and non-tertiary (non-cooling) centres, although the neonates recruited from non-tertiary centres will be transferred to a tertiary centre after randomisation.

### Patient identification and screening

All babies born at or after 36 weeks of gestation and requiring prolonged resuscitation at birth (defined as continued resuscitation at 10 min after birth or 10-minute Apgar score less than 6) or those with severe birth acidosis (defined as any occurrence of: pH less than 7.00 or base deficit *≥* 16mmol/l in the cord or baby’s gas sample within 60 min of birth) and admitted to the neonatal unit will be screened for eligibility.

All neonates meeting the screening criteria will have a neurological examination using expanded modified Sarnat staging by a trained and certified examiner at the time of admission and after one hour of age. The expanded modified Sarnat staging has additional criteria for diagnosis of neonates with mild HIE. A video recording (around 5 to 10 min) of this examination will be obtained in all cases.

Parental consent to use this video recording for research and transfer to the central team at Imperial College for quality assurance will be obtained at a later stage. If parental consent is denied, then the videos will be deleted. Babies with an abnormal neurological assessment will be started on continuous aEEG monitoring and will be recruited if they meet the trial inclusion criteria.

### Inclusion criteria

All babies born at or after 36 weeks of gestation with a birth weight of 1800 g or more with birth acidosis or requiring resuscitation at birth will be screened for eligibility. Parents will be approached for consent if the baby meets all the three (A + B + C) criteria below:

A) Evidence of intra-partum hypoxia-ischemia defined as any of: Apgar score of less than six at 10 min after birth or continued need for resuscitation at 10 min after birth or severe birth acidosis defined as any occurrence of: pH less than 7.0 or Base deficit *≥* 16mmol/l in a cord or baby gas sample within 60 min of birth.
B) Evidence of mild HIE defined as: two or more abnormal findings in any of the six categories of the expanded modified Sarnat examination (level of consciousness, spontaneous activity, posture, tone, primitive reflexes, and autonomic nervous system) but not meeting the diagnosis of moderate or severe HIE on a standardised examination performed by a certified examiner between 1 and 6 h of age.
C) Normal amplitude, with or without sleep wave cycling, on the aEEG performed for at least 30 min between 1 and 6 h of age. Normal amplitude will be defined as upper margin of the aEEG activity more than 10 microvolts and the lower margin more than 5 microvolts on a single channel aEEG.

### Exclusion criteria

- Neonates who meet the BAPM criteria for whole-body hypothermia for moderate/severe HIE.
- Neonates without encephalopathy defined as less than two abnormalities on structured neurological examination.
- Neonates with major congenital or chromosomal anomalies identified prior to randomisation.
- Neonates with birthweight less than 1800 g or gestational age less than 36 weeks at birth.
- Neonates who received muscle relaxation, or anti-seizure medications prior to neurological assessment that impacts the neurological examination.
- Neonates with moderate or severe background voltage abnormalities or seizures on aEEG.
- Neonates already enrolled in interventional studies.

### Randomisation and trial intervention

If the neonate meets the trial inclusion criteria, the availability of a cooling device and intensive care cot space at a cooling centre will be checked before approaching parents for trial participation. Once parental consent is obtained, babies will be randomised to whole-body hypothermia or normothermia within 6 h of birth, using a web-based program developed by Sealed Envelope private limited, London. The randomisation will be performed using minimisation to balance the treatment allocation by site and severity of encephalopathy within mild encephalopathy.

Initial assessment and randomisation (and initiation of whole-body hypothermia or normothermia) will occur at the hospital of birth. The babies in both arms, who are born at a non-cooling centre (Local Neonatal Unit (LNU) or Special Care Baby Unit (SCBU)) will be then transferred to the nearest neonatal intensive care (NICU) cooling centre for continued care.

### Whole body hypothermia (intervention group)

Whole-body hypothermia (33.5 *±* 0.5 °C) will be initiated within 6 h of birth and continued for 72 h using a servo-controlled cooling machine at the hospital of birth. Neonates born at a non-cooling centre will be transferred to an NICU cooling centre for continued care. Passive cooling methods will not be allowed. After 72 h of whole-body hypothermia at 33.5 *±* 0.5 °C, the baby will be rewarmed at 0.5 °C per hour to reach 37.0 *±* 0.5 °C over 6 to 8 h.

### Normothermia (Control group)

The rectal temperature will be maintained at 37 *±* 0.5 °C using servo-controlled incubators with specific attention to temperature management for preventing iatrogenic hyperthermia. Rectal temperature will be recorded, as in the whole-body hypothermia group.

Babies in the control group who develop seizures and progress to moderate HIE between 6 and 24 h may be treated with whole-body cooling for 72 h as clinical care, at the discretion of the clinical team. It is estimated that around 5% of the neonates in both groups may develop seizures after 6 h of age.

### Sedation

Pre-emptive use of narcotic infusions, a common practice during whole-body hypothermia, is often a major concern for parents. Secondary analysis of the NICHD Neonatal Research Network hypothermia trial and the Magnetic Resonance Biomarkers in Neonatal encephalopathy (MARBLE) study have reported opioid sedation increased hospital stay and duration of ventilation and had no neurodevelopmental benefits [26–29]. Hence, use of pre-emptive sedation during whole-body hypothermia will be minimised unless the neonate is distressed with persistent tachycardia and with documentation of pain (NPASS) scores.

### Monitoring and care in both groups

Babies with breathing difficulties or apnoea will have appropriate support with non-invasive (CPAP/high flow) or invasive ventilation. All babies will have continuous aEEG monitoring (minimum one channel; 3 electrodes) for at least 48 h after birth. Continuous monitoring using aEEG for the first 88 h is the current standard care for babies undergoing whole-body hypothermia in the NHS. All neonates will have monitoring of physiological and laboratory parameters as clinically indicated. Enteral milk feeds will be administered in both groups and increased as tolerated as per local unit protocols.

### Training and certification on neurological assessment

The success of the trial recruitment depends on training and certification of all front-line clinical staff at the recruiting sites on expanded modified Sarnat staging (Table 1) [30]. The training will involve a lecture explaining the Sarnat staging, animated and actual videos of the neurological examination as well as post-training evaluation and certification. The entire training can be completed over 90 min using a fully virtual platform. The training will be repeated on an annual basis. In addition, monthly feedback on the neurological assessments of all screened and recruited neonates will be provided to each site to ensure quality assurance.

**Table 1:**
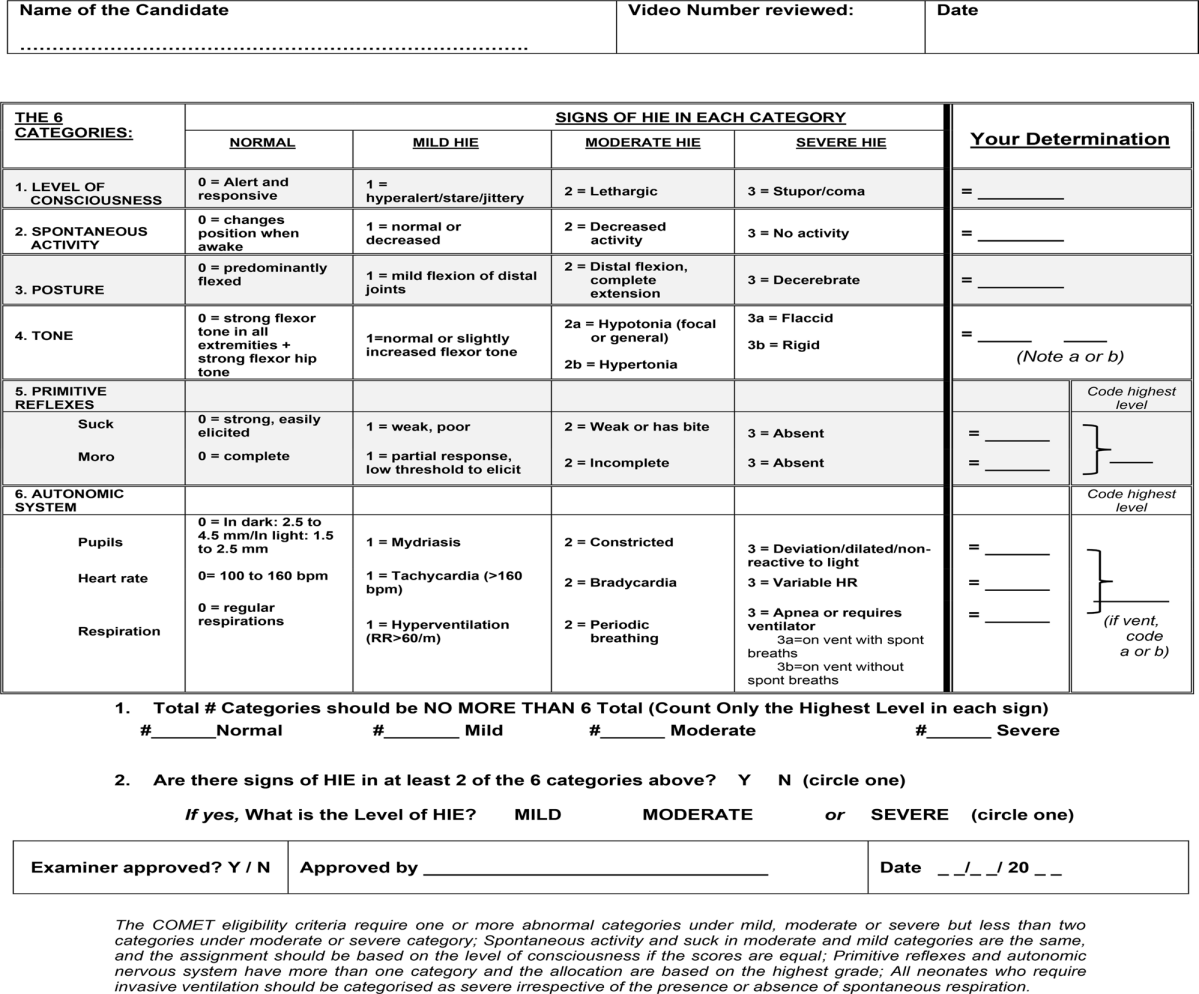
Certification form on the expanded modified Sarnat staging based on the NICHD neonatal research network hypothermia trials [22]

### Withdrawal criteria

Neonates will be withdrawn from the study if either parents withdraw consent or due to the clinician’s decision at any time. A withdrawal form will be filled in and consent will be obtained for use of the data collected up to the withdrawal from the trial. Discontinuation of the study intervention for a serious adverse event will be at the discretion of the attending physician in consultation with the site principal investigator. The neonate will continue to be part of the study as per the intent-to-treat principle.

### Assessment and follow-up

The follow-up assessment will be done when the recruited babies are 24 (± 2) months of age, by a central team of 2 to 4 examiners, masked to the trial group allocation. The assessment will be carried out using the Bayley Scales of Infant and Toddler Development IV. This is a formal examiner administered age-standardised test that assesses development in three domains: cognition, language, and motor development. In addition, all neonates will have a detailed neurological examination, including Gross Motor Function Classification System (GMFCS) for cerebral palsy, vision, and hearing assessment.

Each assessor will be trained by the test publisher (Pearson UK) and certified against the COMET gold standard examiner prior to the assessments being conducted. The examiners will be re-certified against the gold standard examiner annually to reduce interobserver variability to 10%. Vision and auditory status of neonates will be collected as part of the medical history. The follow up visit will be scheduled in close consultation with the parents, either at the local hospital or at home. Appropriate travel expenses will be provided for families for the visit. Travel expenses will also be provided/reimbursed to research team members who must travel to perform the follow up examination.

### Primary outcome

The primary outcome is the mean Cognitive Composite Scale score from the Bayley IV examination at 24 (*±* 2) months of age. The RCTs of hypothermia for moderate or severe encephalopathy have used the composite outcome of death or disability because of high mortality rates, whereas mortality is expected to be low in mild encephalopathy. Therefore, a continuous numerical developmental score among survivors will be more robust to detect treatment effects than categorical outcomes and hence was selected as the primary outcome.

Babies who die (the mortality rate is expected to be less than 1% in mild HIE) or who cannot be assessed with the Bayley-IV due to severe disability (also anticipated to be low in frequency) will be allocated a Cognitive Scale Composite score one point below the basal test score (i.e., score of 54) [31, 32].

If the child is too tired to co-operate with the Bayley assessment at the time of the original appointment, the assessment will be re-scheduled and performed at a place more suitable for the child, for example at home, within the window period of assessment.

### Secondary outcomes

Outcomes assessed during neonatal hospitalisation:

1. Seizures (clinical and aEEG confirmed).
2. Duration of intensive care defined as number of days of neonatal intensive care.
3. Duration of hospital stay defined as the total number of days of inpatient care in a neonatal unit.
4. Duration of mechanical ventilation defined as number of hours on invasive ventilation through an endotracheal tube.
5. Duration of inotropic support defined as total number of hours on inotropic support.
6. Bloodstream or cerebrospinal fluid positive infection defined as isolation of a pathogenic organism from blood or cerebrospinal fluid along with a clinical diagnosis of sepsis, at any time during neonatal hospitalisation.
7. Thrombocytopenia or coagulopathy requiring transfusion of blood products.
8. Successful breastfeeding (at hospital discharge).
9. Brain injury scores on conventional magnetic resonance imaging.

Longer term secondary outcomes assessed at 24 (*±* 2) months include:

1. Survival without any neurological impairment defined as a score of *≥* 85 in all Bayley-IV domains (motor, language, and cognitive), normal neurological examination with no cerebral palsy (Gross motor function classification system score 0), no hearing or visual impairment (as reported by parents), and no seizure disorder.
2. Preschool Child Behaviour Checklist (CBCL 1½-5) will be completed by parents at the 24(*±* 2) month assessment to provide a standardised measure of children’s behavioural outcomes on scales that assess internalizing and externalizing behaviour problems and a Total Problems Scale. Mean standardised T-scores on each scale will be compared between groups. The CBCL checklist will be completed after the Bayley IV assessments.
3. Cerebral palsy with a gross motor function classification system score (GMFCS) of 2 or more.
4. Head circumference of less than 2 standard deviations.

### Exploratory studies

1. Mechanistic studies: Overall, approximately 2.5 ml of blood will be collected for mechanistic studies in all babies: (i) 1.0 ml will be collected within 0–6 h of age, (ii) 0.5 ml will be collected at 48 (*±* 4) hours of age, and (iii) 1.0 ml will be collected at around 84 (± 4 h) hours of age.
2. Magnetic resonance spectroscopy: In centres with facilities for undertaking proton magnetic spectroscopy using harmonised sequences for quantification of thalamic n-acetyl aspartate (NAA), these sequences will be acquired at the time of routine clinical MR scanning.

## Study management

The day-to-day management of the study will be co-ordinated through the Centre for Perinatal Neuroscience, Imperial College London. The trial management group (TMG) will oversee all aspects of the day-to-day running of the study, and will consist of the investigators, trial manager, trial research fellow and other COMET trial staff based at the Centre for Perinatal Neuroscience, Imperial College London. TMG will hold a monthly teleconference of all COMET investigators for the entire duration of the trial to discuss the data quality and recruitment.

### Data collection tools

Prior to the start of recruitment, a manual of procedures (MOP) will be developed providing details of the protocol design and procedure and definitions of each data variable, and procedures for data lock. All study personnel entering the data (research nurses and site principle investigators) will be trained and certified during site initiation and names will be documented in the delegation log.

### Sample size calculation

The Bayley-IV Cognitive Scale Composite score has a normative mean of 100 and SD of 15. To detect a clinically important minimum difference of 5 points (0.3 SD), at a 0.05 significance level and 90% power, we would need 191 neonates per group, 382 in total. This increases to 426, after allowing for a conservative 10% drop-out rate (Table 2). The total duration of the trial is 66 months which will include a six-month trial set up period, 30 months of recruitment, and outcome assessments at the age of 24 (*±* 2) months.

**Table 2:** Sample size calculation

Size of group difference	Total study sample size	
	90% power	80% power
4 points	660	494
5 points	426	318
6 points	296	224
7 points	218	166
8 points	168	128
9 points	134	100
10 points	110	84

The implication of changing the power of the study and the size of outcome differences between groups has been examined and is shown in the subsequent table. This shows the total sample size required in both groups combined, after allowing for a 10% drop-out rate (Table 3).

**Table 3:** Effect of drop out on study power

Drop-out rate	Study power
10%	90.0%
12.5%	89.4%
15%	88.5%
17.5%	87.7%
20%	86.5%

The assumed attrition rate of 10% is conservative, as we have consistently obtained > 97% follow up at 18 to 22 months in previous trials performed in the UK. The implications of a higher drop-out rate upon the power of the trial are shown below. If the drop-out rate is 20%, the study would still have an 87% power to detect a 5-point difference between groups.

### Statistics and data analysis

The primary outcome is the Cognitive Scale Composite score from the Bayley-IV examination at 24(*±* 2) months. Based on previous experience the scores are expected to be approximately normally distributed, and thus a two-sample t-test will be used to compare between groups. The mean difference in outcome between groups will be reported, along with a corresponding confidence interval. If the outcome scores are not normally distributed, an appropriate data transformation will be explored, or alternatively a non-parametric test (Mann-Whitney test) may be utilised.

Secondary outcomes are both short term (in hospital) or longer term (at 24 months). Continuous secondary outcomes will be analysed using the unpaired t-test if normally distributed, or the Mann-Whitney otherwise. The Chi-square test or Fisher’s exact test will be used to compare categorical outcomes between groups. For each outcome, a point-estimate of difference between groups will be reported, alongside a corresponding confidence interval.

### Health economic evaluation

A prospective health economic evaluation will be embedded within the trial design. The health economic evaluation will adopt a UK NHS and Personal Social Services perspective in accordance with the National Institute for Health and Care Excellence (NICE) Reference Case [33].

Primary research methods designed will mirror those applied in our economic evaluation conducted as part of the TOBY trial [34], and will include capital and non-capital costs of the whole-body hypothermia system, transport costs to tertiary cooling centres, and length of hospital stay. Downstream resource consequences until hospital discharge, including duration and intensity of care provided, will be captured through trial case report forms. In addition, online economic questionnaires completed by parents at 6, 12, 18, and 24 months will document post-hospitalisation resource utilisation. Unit costs for post-hospital discharge resource inputs will largely be derived from national reference tariffs, although primary research that uses established accounting methods may also be required.

The cost-effectiveness of whole-body hypothermia will be expressed in terms of incremental cost per unit change in the cognitive composite score of the Bayley-III examination (cost-effectiveness analysis). Bivariate regression of costs and consequences, with multiple imputations of missing data, will be conducted to generate within-trial estimates of incremental cost-effectiveness associated with whole body cooling. Sensitivity analyses will be undertaken to assess the impact of areas of uncertainty surrounding components of the economic evaluation. The sensitivity analyses will include re-estimation of cost-effectiveness based on cases with complete data, and re-estimation of cost-effectiveness assuming a broader societal perspective. The latter will incorporate direct costs borne by families and friends, for example, travel costs, economic values for informal care provided by family and friends, and economic values associated with productivity losses; the values of these broader resource consequences will be informed by responses to questions in the online parent-completed questionnaires. Cost-effectiveness acceptability curves will be used to show the probability of cost-effectiveness of whole-body hypothermia at alternative cost-effectiveness thresholds with plausible economic values associated with improvements in cognition informed by a literature review.

Decision-analytic modelling, drawing upon our previous decision model developed as part of the TOBY trial [34], will be used to estimate the long-term cost-effectiveness of whole-body cooling with cost-effectiveness expressed in terms of incremental cost per quality-adjusted life year (QALY) gained (cost-utility analysis). Parameter inputs into the decision-analytic model will be informed by our earlier research conducted as part of the TOBY trial, supplemented by targeted literature searches. Approaches for characterising uncertainty, heterogeneity, and distributional effects within the economic evaluation will adhere to the recommendations of the NICE reference case [33]. The economic evaluation will be prospectively planned and detailed within a ‘Health Economic Analysis Plan’ and signed off by the Trial Steering Committee.

### Recordings and reporting of SAEs, SARs and SUSARS

Safety outcomes will consist of measurements of Adverse Events (AEs) and Serious Adverse Events (SAEs) (see section below). If there are sufficient numbers of AEs and SAEs, the Chi-square test or Fisher’s exact test will be used to compare the number of patients with these outcomes between groups. The Mann-Whitney test will be used to compare the number of AEs/SAEs between groups. A list of individual AEs will be reported in each group. All analysis will be performed on a modified Intention to Treat (mITT) basis, using patients with valid outcome data in the analysis. Neonates will be analysed in the groups to which they were randomised (intent-to-treat analysis), regardless of the treatment received.

Monitoring for adverse events of whole-body hypothermia in the intervention group during the intervention period and both groups during the entire length of hospital stay will be conducted by evaluating events described in the secondary outcomes. An additional safety measure will be the appointment of an external Independent Data Monitoring Committee (IDMC) where the progress of the trial and adverse events (AE) will be closely monitored at 4 to 6 monthly intervals, masked to the allocation. The IDMC charter will be finalised and signoff before the start of recruitment.

**The adverse events** will include persistent metabolic acidosis, thrombosis, major bleeding, perforations/ulcerations/bleeding from the rectal probe, hyperglycaemia, hypoglycaemia, necrotising enterocolitis (NEC), thrombocytopenia requiring platelet transfusions, coagulopathy requiring blood products, loss of skin integrity, and hypotension requiring more than 2 inotropes.

**Serious adverse events (SAE)** will include mortality, major cerebral bleeds on MRI, pulmonary bleeds, PPHN requiring inhaled nitric oxide or extra-corporeal membrane oxygenator (ECMO), or any other clinical event the investigators deem as life threatening. Additional SAE reports may be requested (e.g., monthly) throughout the course of the study. Safety will be assessed by the frequency of SAE, and total number of events per baby.

### End of study

The end of the trial will be notified to the sponsor. The date of the 24 (+ 2) months follow-up of the last patient undergoing the trial will be considered as the end of the current trial.

### Future directions

Additional funding will be sought for assessment of childhood outcomes at a later stage.

### Archiving

Archiving will be authorised by the Sponsor following submission of the trial report. All essential documents will be archived for 10 years after completion of the trial. Authorisation will be taken regarding the destruction of essential documents.

## Discussion

We describe the protocol of the COMET trial, a phase III randomised controlled trial of whole-body hypothermia mild encephalopathy. The COMET trial will establish the safety and efficacy of whole-body hypothermia for mild HIE, inform national and international guidelines, and will establish uniform practice across the NHS and other high-income countries. It will also provide an economic case for the NHS, if whole-body hypothermia is beneficial. Alternatively, whole-body hypothermia treatment will be discontinued for babies with mild HIE if it is found to be ineffective or unsafe, again leading to cost savings. In the absence of a clinical trial, whole-body hypothermia will be increasingly used for this population, and safety and efficacy will remain unknown. An additional downstream effect of the COMET trial is a national standardisation of neurological assessment based on expanded modified Sarnat Staging.

The COMET trial design was informed by a COMET pilot randomised controlled trial data and extensive discussion with parents of babies with mild HIE [35]. A total of 101 neonates with mild HIE from six tertiary neonatal intensive care units in the UK and Italy were recruited in the COMET pilot trial over 3 ½ years. The neonates were randomised based on the age at randomisation. Those aged less than 6 h at the time of recruitment were randomised to either normothermia or therapeutic hypothermia for 72 h at 33.5 °C (early randomisation cohort). The neonates with mild HIE who were already started on therapeutic hypothermia as part of clinical care were randomised to either rewarming at 48–72 h of age (late randomisation cohort). Although the injury scores on conventional MR were similar across the groups, the mean (SD) [NAA] level was higher in the normothermic group compared with 48 h and 72-hour hypothermia groups. Seizures after six hours of birth occurred in 2.9%, 3.2% and 5.5% of the normothermic, 48 h and 72-h hypothermia groups [35].

Thalamic [NAA] was higher in neonates with mild HIE who were rewarmed after 48-hour hypothermia than those who had 72 h of hypothermia. Most neonates in the late randomisation cohort were born at non-cooling centres and were initiated on whole-body cooling soon after birth without adequate neurological assessment or aEEG examination before transfer to a cooling centre [35]. These observations suggest that involvement and training of frontline clinicians in non-cooling centres on neurological assessment is of critical importance in clinical trials of whole-body hypothermia. Furthermore, careful monitoring including continuous aEEG of all neonates with mild HIE is important for prompt detection and management of seizures occurring after 6 h of age.

The views of parents differed from heath care professionals about the care in the normothermic control arm. Parents preferred that all neonates with mild HIE should be transferred to tertiary centres for neurological monitoring in view of the risk of brain injury and potential impact on later life. They did not consider transient separation from their baby as a major concern, in the context of brain injury. Clinicians on the other hand were concerned about overburdening of tertiary centres from the additional admissions, costs of neonatal transport and considered parental separation as a major issue. The COMET pilot trial data suggest recruitment to the trial is unlikely to increase the number of neonates currently being offered cooling therapy most neonates with mild HIE are currently being cooled in the NHS without adequate neurological assessment and transferred to cooling centres. Hence, in the COMET trial, we intend to use a network work approach involving both tertiary and non-tertiary centres and develop a robust program for training and certification of neurological assessment.

### Dissemination

The success of the COMET RCT depends on a large number of neonatal junior doctors, nurses and neonatologists recutting neonates to the trial. Credit for trial participation will be given to all who have collaborated, including all local co-ordinators and collaborators, members of the trial committees, the COMET trial Co-ordinating Centre and trial staff. Authorship at the head of the primary results paper will take the form “[name], [name] and [name] on behalf of the ‘The COMET trial Collaborative Group’”. All contributors to the trial will be listed at the end of the main paper, with their contribution identified. The trial data will be discussed with parents of all neonates who participated in the trial before publication. The data will be presented at various national and international conferences, in addition to peer reviewed open access publications.

### Independent Data Safety Monitoring Committee

Dr Christopher Partlett, Nottingham Clinical Trials Unit (Chair), Dr Amarnath Bhide, St George Hospital, Dr Ajay Sinha, Royal London Hospital.

### Steering committee independent members

Mr Matthew Dodd, London School of Hygiene & Tropical Medicine; Professor Samir Gupta, Durham University; Associate Professor Hema Mistry, Warwick Clinical Trials Unit Health Services Research Institute, University of Warwick; Ms Cristina Costa, PPI Representative; Dr Jethro Herberg, Imperial College London; Ms Gaby Richter, PPI Representative; Professor Jayaprakasan, Kannamannadiar, Derby Hospitals NHS Foundation Trust.

**Fig. 1:**
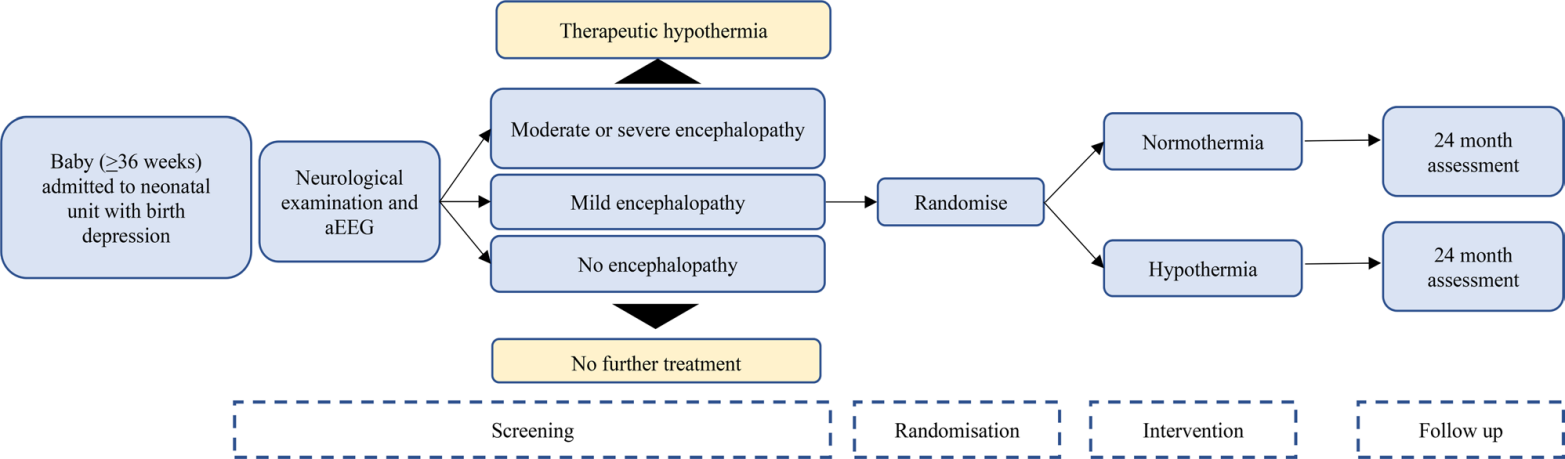
Study Flow Diagram

## Data Availability

Direct access will be granted by Dr Sudhin Thayyil to authorised representatives from the Sponsor, host institution, and the regulatory authorities to permit trial-related monitoring, audits, and inspections in line with participant consent.
